# Connections between Orthopedic Conditions and Oxidative Stress: Current Perspective and the Possible Relevance of Other Factors, Such as Metabolic Implications, Antibiotic Resistance, and COVID-19

**DOI:** 10.3390/medicina58030439

**Published:** 2022-03-17

**Authors:** Bogdan Huzum, Alexandrina Stefania Curpan, Bogdan Puha, Dragomir Nicolae Serban, Bogdan Veliceasa, Riana Maria Necoara, Ovidiu Alexa, Ionela Lacramioara Serban

**Affiliations:** 1 Department of Orthopaedic and Traumatology, Faculty of Medicine, “Grigore T. Popa” University of Medicine and Pharmacy, 700115 Iasi, Romania; bogdan.huzum93@gmail.com (B.H.); puhab@yahoo.com (B.P.); velbogdan@yahoo.com (B.V.); ovidiu_alexa@yahoo.com (O.A.); 2 Department of Physiology, Faculty of Medicine, “Grigore T. Popa” University of Medicine and Pharmacy, 700115 Iasi, Romania; ilserban1@yahoo.com; 3 Department of Biology, Faculty of Biology, “Alexandru Ioan Cuza” University of Iasi, Carol I Avenue, 20A, 700554 Iasi, Romania; 4 Radiology-Imaging Clinic, “Sf. Spiridon” Clinical Emergency Hospital, 700111 Iasi, Romania; riana.necoara@gmail.com

**Keywords:** oxidative stress, orthopedic, COVID-19, antibacterial resistance, osteoarthritis, osteoporosis, orthopedic surgery

## Abstract

The general opinion in the literature is that these topics remain clearly understudied and underrated, with many unknown aspects and with controversial results in the respective areas of research. Based on the previous experience of our groups regarding such matters investigated separately, here we attempt a short overview upon their links. Thus, we summarize here the current state of knowledge regarding the connections between oxidative stress and: (a) orthopedic conditions; (b) COVID-19. We also present the reciprocal interferences among them. Oxidative stress is, of course, an interesting and continuously growing area, but what exactly is the impact of COVID-19 in orthopedic patients? In the current paper we also approached some theories on how oxidative stress, metabolism involvement, and even antibiotic resistance might be influenced by either orthopedic conditions or COVID-19. These manifestations could be relevant and of great interest in the context of this current global health threat; therefore, we summarize the current knowledge and/or the lack of sufficient evidence to support the interactions between these conditions.

## 1. Introduction

Osteoarthritis (OA), one of the most prevalent forms of arthritis, is a degenerative disorder of the joints. It occurs in 40% of the population above the age of 70 years old. The progression of OA is associated with worsening pain and joint stiffness, with the knees being the most frequently affected by OA [1]. OA chronically involves the entire joint, going from focal cartilage loss to structural alterations of ligaments and muscles, in a progressive manner [2,3]. The multifactorial origin of OA comprises various theories, such as: the “storm” of inflammatory cytokines; the mechanical injury of the cartilage or high-impact physical activity and apoptosis of chondrocytes; the cellular senescence, which eventually leads to increasing levels of reactive oxygen species (ROS). In fact, each of these processes results in an increased formation of ROS as byproducts [3,4,5].

Besides aging, other risk factors include obesity, crystal deposition, polyarticular diathesis, genetic predisposition, gender, and metabolic disorders. As obesity and metabolic disorders are risk factors, OA can often be present alongside diseases like diabetes mellitus and cardiovascular diseases [5,6]. Since obesity is a major issue in modern society and with the life expectancy increasing every year, OA will also become a more prevalent disorder, even in younger populations [6].

One of the underlying mechanisms of the development and progression of OA, as above mentioned, is apoptosis of chondrocytes, which in turn determines cartilage degeneration and bone thickening [7]. Chondrocytes are the only cell type in cartilages and have the purpose of maintaining cellular homeostasis under oxidative stress [8]. In the case of degenerative disease, alongside apoptosis, autophagy may be closely associated and decreased in OA, as one study suggests; based on the analysis of the changes in p62 and cleaved caspase 3 expression, which indicated a disrupted autophagy flux [3].

Aging is also a primary cause for bone tissue loss, in almost every population, with a debut near the age of 40 and acceleration after the age of 60. The clinical manifestations of bone tissue loss include increased bone porosity, decreased bone mineral density and altered bone microarchitecture, which eventually increases bone fragility and the proneness to fractures. Bone tissue loss determined by age is one of the major causes of osteoporosis in the older population [9,10,11].

Bone density and strength are maintained by two types of cells, osteoblasts and osteoclasts. Aging affects the bone cells themselves. More precisely, during aging the number and activity of osteoblasts are decreased. Studies on animal models of osteoporosis after spinal cord injury have revealed a reduced viability of osteoblasts, similar to that found in postmenopausal patients, and also altered responses to hormones such as estrogen. Such aspects support the importance of the malfunction of osteoblasts in the pathogeny of osteoporosis [11].

Even if osteoporosis is generally a disease associated with age, young people tend to suffer from secondary osteoporosis following treatment with glucocorticoids, with nearly 1/5 of patients suffering fractures within the first 12 months of treatment. Previous in vitro studies have illustrated that glucocorticoids possess the power to induce apoptosis of osteoblasts and osteocytes, with the same effect being observed in vivo. There are several hypotheses as to how glucocorticoids manage to activate apoptosis in these specific cells, but the in vivo and in vitro studies have not managed to elucidate the full mechanism yet. However, based on these studies, one of the proposed mechanisms involves increased ROS in the bone tissue [12].

Another common orthopedic condition is rotator cuff tear. This may be due to external events, such as trauma, or to internal factors, such as inflammation, aging, and degeneration. Studies of rotator cuff tear on animal models have revealed thinner collagen fibers and disarrangements, but increased fibrocartilage in the rotator cuff and loss of cellularity [13].

Chronic inflammation also highlights the profile of rheumatoid arthritis (RA) which is an autoimmune disease affecting 1–2% of the population. As an autoimmune disease, RA is described through immunological aberrations and chronic inflammation and its etiology is thought to be comprised of environmental impacts (such as viral infections and gut bacteria, smoking and dietary components) on genetically susceptible individuals, as the heritability of RA is about 50–60% [14].

We performed an initial literature search in July 2021 and included articles published after the year 2000, with a focus on the most recent relevant ones. The articles were found by using, under different combinations, keywords such as: oxidative stress, orthopedic, COVID-19, antibacterial resistance, osteoarthritis, osteoporosis, and orthopedic surgery. The search was performed by accessing MEDLINE, Hindawi, and Google Scholar databases. Subsequently, articles were selected by the presence of the keywords in the title and abstract and their connection to orthopedic conditions, COVID-19, secondary bacterial infections and oxidative stress. We excluded articles not directly related to the subject of interest, such as those regarding COVID-19 patients’ management or risks, or those regarding neurodegeneration.

## 2. Metabolic Implications

OA can be categorized based on various parameters, but metabolic OA is rather new and interesting in its nature. This one highlights the important roles played by metabolism and also how metabolic interactions and disturbances may affect OA, especially when they are present in the form of metabolic syndrome (MetS). MetS goes from disturbances in the glucose metabolism to comorbidities such as obesity, arterial pathology and dyslipidemia; it finally triggers type 2 diabetes and cardiovascular diseases, illustrated by higher insulin and C-peptide levels. Studies suggest that MetS and oxidative stress co-exist and that they are both underlying mechanisms for OA. The severity of OA has been correlated with both LDL-cholesterol and with oxidized LDL, and LDL is generally susceptible to ROS. Interestingly, MetS has been found to be more prevalent among OA patients with cholesterol accumulating in OA chondrocytes. One study indicated that impaired glucose metabolism might be correlated with OA [6].

Aerobic and anaerobic glycolysis coexist in the normal chondrocytes subjected to the physiological conditions of normal oxygenation. The reactions involved in these pathways are usually reversible, with the exception of glucose import, hexokinase, phosphofructokinase, and lactate efflux. Glucose involving mechanisms are thought to participate in the pathogenesis of OA, because anaerobic glycolysis is increased in OA. This affects the tricarboxylic acid cycle and oxidative phosphorylation, because less pyruvate is being converted to acetyl-CoA. One study has illustrated hyperlipidemia as a possible risk factor for hand OA, as a possible association between omega-6 polyunsaturated fatty acids (PUFAs) and synovitis was observed. Omega-6 PUFAs mediate ROS production and chondrocytes apoptosis through nicotinamide adenine dinucleotide phosphate oxidase 4 (NADPH oxidase 4; NOX4). Another study has pointed out a possible correlation between an increased cholesterol intake and OA on the medial side of the joint, while decreasing it in vivo attenuated the severity of the disease [5].

MetS-associated OA involves alterations of chondrocytes’ metabolism, as follows. In the situation of OA and inflammatory conditions, chondrocytes experience disturbed mitochondrial function, chondrosenescense and enhancement of the glycolytic pathway, as a consequence of the pathological shift in the metabolic homeostasis [5]. Interestingly, several studies have also reported acute hypocalcemia as a frequent finding in infections with viruses causing severe acute respiratory syndrome (SARS) [15,16].

## 3. Oxidative Stress and COVID-19

Orthopedic conditions are an ongoing worldwide problem, but this happens in parallel with an ever-increasing life expectancy and with the new threats brought along with the various complications. Of course, these include the COVID-19 pandemic, which is by itself a dangerous disease for the older generation. For example, it would be interesting to see some approaches, regarding the complications, when a patient suffers from an orthopedic disorder, such as osteoarthritis or osteoporosis, and also from COVID-19.

Oxidative stress has also been recently proposed as a key player in COVID-19. The mechanism of action of COVID-19 (SARS-CoV-2; severe acute respiratory syndrome coronavirus 2) is by accessing the human respiratory epithelial cells through angiotensin converting enzyme 2 (ACE2). ACE2 cleaves angiotensin II (Ang II) and induces vasodilation.

Recent studies have shown that the affinity of SARS-CoV-2 for ACE2 is one to two times higher than that of the first SARS-CoV faced by humanity. As ACE2 may have protective functions, the possible decrease in its level is unavoidably correlated to adverse clinical phenotypes. Oxidative stress and inflammatory responses are determined by SARS-CoV-2 binding to ACE2, thereby reducing its bioavailability. This would promote Ang II interaction with angiotensin receptors (AT1 and AT2), but AT2 also activates NADPH oxidase (NOX). These are some of the features associated with the oxidative stress present in COVID-19: faster heart rate, cold extremities, acidosis, high lactate, and hyperbilirubinemia [17,18] An overexpression of ACE2 as a means for lowering oxidative stress is supported by the fact that hypertension has been correlated as a side effect of oxidative stress. The affinity of the virus for the ACE2 receptor is intensified by the oxidation of cysteine residues [18]. Available studies report connections among oxidative stress, the severity of the COVID-19 infection, and several risk factors such as older age, sex, ethnicity, hyperglycemia, and obesity.

This virus infects type II pneumocytes, which contain a large number of mitochondria, using acetyl-CoA in the metabolism of fatty acids and it was proposed that hyperoxia induces ROS generation [19]. As with the elderly and people with diabetes, hypertension and cardiovascular disease are already in an oxidative state, it might explain why these categories are more susceptible to severe forms of COVID-19 infections [20].

Interestingly, there is a depletion of endogenous glutathione in severe cases of infection, which might be the cause of the oxidative homeostasis shift towards imbalance, leading to aggravated lung inflammation and eventually to respiratory distress, multiorgan failure and death. One of the main reasons why men are more susceptible to severe forms of COVID-19 is that men have lower plasma levels of glutathione compared to women, which makes them more prone to oxidative stress and inflammation. Smokers are also more susceptible to oxidative stress and lung inflammation while infected with COVID-19 because cigarette smoke is known to deplete the glutathione reserves. Based on these studies, several others have suggested that higher levels of glutathione might improve the response to viral infections, as it protects the host immune cells [21]. Age is also associated with reduced levels glutathione (GSH); its concentration decreases with the age increase [18].

Recent studies suggest that ROS may facilitate viral entry and promote viral infectivity in an oxidative environment, contrary to the popular opinion that ROS are involved in eliminating pathogens [22]. The viral infection triggers ROS production and significantly affects the production of oxidizing agents alongside antioxidant enzymes, and at acute levels it promotes viral growth, while at chronic levels it participates in tissue damage. SOD and H_2_O_2_ are produced by neutrophils and macrophages as a defense against viruses, to limit their multiplication [23,24]. The activated phagocytes are a main source of endogenous oxidative species as a result of the cytokine storm.

The free radicals are capable of inducing oxidative lesions of the genetic material, triggering cell lethality, mutagenesis, carcinogenesis and apoptosis. Another oxidative stress marker that is altered in COVID-19 infections if MDA, which has been observed in several studies to be increased in the blood plasma during the viral infection [25].

## 4. Orthopedic Conditions and COVID-19

After we conducted our literature search, thus, to the best of our knowledge, we identified limited articles approaching orthopedic conditions in regard to SARS-CoV-2 infection, except for rheumatoid arthritis (RA) as an autoimmune disorder. One of the main reasons for the lack of literature on this theme is the fact that most surgeries requiring bone replacement have been postponed during the pandemic, as a major percentage of orthopedic patients are elderly and with at least another three comorbidities. The identified risks are summarized in Table 1.

RA patients constitute a high risk group for COVID-19 infection due to the glucocorticoid treatment and the premature aging of the immune system, which exposes the organism to infections [26]. Glucocorticoid treatment influences the risk in a dose-dependent manner, with prior treatment increasing the prevalence, while patients with rheumatoid diseases have the highest risk for hospitalization amongst autoimmune diseases [27] and an even higher risk for ICU admission and mechanical ventilation [28,29,35,41,51] compared to the general population. One study has identified a higher risk of venous thromboembolism in RA patients that have contracted the infection, with glucocorticoids only increasing the risk of adverse effects [58], whereas systemic therapies did not influence it [34], nor did other classes of disease-modifying antirheumatic drugs [64]. On the other hand, one study has suggested that respiratory viral infections might be in fact a risk factor for the development of RA [65], but treatments with prednisolone and TNFα inhibitors (TNFis) increase the risk of COVID-19 [36], and the risk of hospital admissions [29] in RA patients with comorbidities such as diabetes. Two studies have found contradicting results; one found no association between RA and COVID-19 in a cohort study in South Korea [31], whereas the other stated that RA patients are more prone to the infection due to the iatrogenic effects of the RA medications [30].

One common complication has been reported in inflammatory reactive arthritis based on the hypothesis of the proinflammatory markers (IL-6 and TNF-*α*) being released in alveolar and musculoskeletal inflammation [61]. Similar to RA, one common post-COVID-19 complication is an early onset of arthritis that can be cured by non-steroidal anti-inflammatory drugs. The manifestations are very similar to osteoarthritis, but it is not a viral arthritis as no viral RNA has been found in the joints and the prevalence is much higher. One possible explanation could be hypocalcemia, vitamin D deficiency, and pro-inflammatory cytokine production, alongside the expression of ACE2 and the disruption of RAS [62], which lead to bone resorption and promote bone loss [59].

One study has illustrated an increased risk of hospitalization and death following SARS-CoV-2 infection in patients with a history of hip [44,46], spine, humerus, and wrist fractures [38], as well as early postoperative mortality [47] and increased hospital stay [37,39], whereas another study has exhibited that in fact the COVID-19 infection increases the prevalence of vertebral fractures, which in turn increases the requirement for mechanical ventilation and the mortality rate [42]. However, one study has identified no significant difference in mortality for osteoporotic upper hip fractures due to the COVID-19 infection both 30 days and 6 months post surgery [48].

Based on our findings, we believe that COVID-19 would at least aggravate or increase the risk of contracting an infection for orthopedic patients. Considering that COVID-19 triggers a cytokine storm upon infection [66], it might aggravate the state of those with autoimmune disorders such as RA.

The pathological implications and relations of these two conditions might represent a subject of interest, especially while orthopedic conditions were and still are neglected in the case of COVID-19 patients, even if this comorbidity might have a big impact [67].

As mentioned in a previous section, diabetes and metabolic syndromes are a risk factor in the case of orthopedic conditions. The situation is similar in the case of COVID-19 and diabetes, either as a comorbidity or a post-infection complication. Diabetes is one of the most frequent comorbidities in people with SARS-CoV-2 and determines a higher rate of hospitalization, pneumonia, and mortality [40]. It is known that diabetes triggers an exaggerated inflammatory syndrome, which suggests that in combination with an autoimmune disease, such as RA, the outcome could be fatal. The decrease in oxygen saturation leads to increased acidosis, which in turn might change the conformation of the virus’ S-protein and lower the antibody affinity and protection [68,69]. Prolonged studies across Europe have reported that populations aged 18 and younger were more likely to receive a diabetes diagnosis after more than 30 days post-infection, whereas in the general population it is a common complication to receive a type 1 diabetes diagnosis or an increase in diabetic ketoacidosis [32]. Several studies have associated hyperglycemia and diabetes with severe outcomes following infection [52,53,54]. Interestingly, at-home glucose-lowering drugs (metformin, dipeptidyl peptidase-4 inhibitors, insulin, sodium-glucose cotransporter 2 inhibitor, alone or in combination with metformin) prescribed for type 2 diabetes mellitus are not significantly associated with mortality or severe outcomes [50], but poor glucose control is associated with poor outcomes [70,71].

## 5. The Role of Oxidative Stress in Orthopedic Conditions

### 5.1. Oxidative Stress and OA

Oxidative stress is a condition characterized by an imbalance between pro-oxidant molecules and antioxidant molecules, caused by increased amounts of ROS. Oxidative stress is also one of the main causes of several orthopedic conditions, such as OA; an excessive pro-oxidant imbalance can eventually cause the death of chondrocytes [72]. In studies on the rat model of OA, it has been noted that ROS lead to oxidative stress, which in turn activates endoplasmic reticulum (ER) stress, along with mitochondrial injury, which then provokes the death of chondrocytes in a cascade manner. During endochondral ossification, the chondrocytes secrete a mass of extracellular matrix. The senescence of the chondrocytes, determined by oxidative stress, is characterized by the degradation of this extracellular matrix. The death of chondrocytes could lead to the degradation of the articular cartilage [7,73]. ER stress can be evidentiated using specific markers, such as glucose-regulated protein-78 and Bcl-associated athanogene-1, which are significantly upregulated in OA [8]. Excessive ER stress also triggers the cleavage of caspase 12 [3]. Oxidative stress has been shown to induce cell apoptosis in both the in vivo and the in vitro models and one study suggests the implications of protein phosphatase 2A in osteoblasts’ death [74].

The chondrocytes’ homeostasis is normally maintained by the mitochondria, disturbed in OA, characterized by inflammation, decreased mitochondrial biogenesis, and increased catabolic activity. These factors, along with telomere-related genomic instability and alterations of the mitochondria structure, lead to oxidative damage. In OA, the reactive oxygen species determine increased inflammatory response, as the activity of mitochondrial complexes II and III are decreased, and also inhibit the synthesis of glycosaminoglycans and type II collagen fibers. ROS-induced chondrocytes apoptosis occurs through the activation of the PI3K/Akt and caspase pathways. In studies measuring the levels of superoxide dismutase (SOD) in OA, the cartilage has shown decreased levels and even cartilage degeneration in the mice missing it altogether [5,75]. Another study illustrated that the levels of ROS and malondialdehyde (MDA) in chondrocytes were significantly higher, which in turn promoted the synthesis of inducible nitric oxide synthase (iNOS) and increased the secretion of nitric oxide (NO). SOD and catalase (CAT) were also higher in the OA chondrocytes compared to normal controls [73]. Another study has noted decreased antioxidant parameters, like SOD and glutathione peroxidase (GPx), but with the exception of CAT, which did not reveal any significant differences. Interestingly, when compared based on sex, CAT values revealed a significant depletion in females vs the respective control group, whereas there was an increase for men [1].

Another study has found extensive staining of nitrotyrosine (a marker of oxidative damage) in the degenerating regions of OA cartilage and a correlation between the intensity of the staining and the histological changes [75]. Several studies have proven the involvement of oxidative stress in the etiology of OA, by administrating antioxidant substances and the check for improvements. One study in particular has chosen to use curcumin as an antioxidant, and they have noted decreased apoptotic chondrocytes and suppressed ER stress [7]. Interestingly, one study was able to demonstrate the implications of oxidative stress in the muscle dysfunction associated with OA development, by observing increased cytokines, increased oxidative stress markers, and increased ROS production [76].

### 5.2. Oxidative Stress and Osteoporosis

Oxidative stress has an effect on bone remodelling and accelerates osteoporosis progress. Oxidative stress is considered one of the initiating factors for the impaired osteoblastic bone, both in the osteoporosis influenced mainly by age and the one determined by menopause [77,78].

As mentioned before, estrogen plays an important role in the antioxidant defense line of lipoproteins and if the levels of estrogen are decreased, the levels of oxygen free radicals and oxidation components will increase [79]. The long-term effect of decreased estrogen levels is characterized by higher levels of oxidative stress, which in turn will result in lipid accumulation and eventually might determine the apoptosis of osteoblasts. Lipid accumulation by itself is responsible for the rise of hydrogen peroxide and superoxide levels, therefore, increasing the oxidative stress that might influence osteoblast activity through the inhibition of differentiation and the promotion of apoptosis [80].

### 5.3. Oxidative Stress and RA

As a chronic inflammatory autoimmune disease, RA is characterized by the presence of the pro-inflammatory cytokines IL-1, tumor necrosis factor alpha and interferon-gamma, which increase the production of NO in the macrophages. NO reacts with O_2_^−^, resulting in peroxynitrite, which is responsible for biomolecular damage, such as protein nitration. The inflamed rheumatoid joint is characterized by: (a) hypoxia, during which dehydrogenase is converted to xanthine oxidase and the affinity for oxygen is increased, generating oxygen ions and H_2_O_2_; (b) acidity, which might promote the reduction in NO_2_^−^ to NO [81]. In autoimmune inflammatory disease there is activation of nicotinamide adenine dinucleotide phosphate oxidase 2 (NADPH oxidase 2; NOX2) and of iNOS, which may determine excessive formation of ROS (including the “reactive nitrogen species”, RNS). The antioxidant pathways are obviously controlled by gene expression and here we mention the nuclear factor erythroid 2-related factor 2 (Nrf2). Certain genes have the “Antioxidant Response Element” sequence in their promotor regulatory regions, and this may be the key for the multiple antioxidant involvements of Nrf2.

Type II collagen is the predominant cartilage collagen. The anti-native type II collagen antibodies occur in less than a quarter of RA patients. However, in significantly more patients, antibodies are produced to better recognize the modified forms of the protein. Regarding oxidative stress markers, one study has identified decreased levels of GPx isoforms 1 and 4 in the neutrophils/lymphocytes of RA patients [14].

## 6. Orthopedic Condition and Antibiotics Resistance

Antibiotics resistance is a serious problem of modern society, as long as antibiotics can be easily acquired as over the counter medicines, despite the efforts of the health organizations to limit the use of antibiotics without prescription. Moreover, this is also because the production of antibiotics is a low-funding area, which raises no interest from the big pharmaceutical companies. Antibiotics are usually cheap, and their effectiveness decreases quite rapidly, thus, permanently requiring newer options, even at the risk of creating more multi-drug-resistant bacteria.

Orthopedics is a field prone to bacterial infections, especially when it comes to bone cements for example; therefore, it is important to better manage antibiotics use and find alternatives that will not produce more damage than wellbeing. Among the most common classes of antibiotics are penicillins (still preferred against *Clostridium*), cephalosporins (capable of preventing cell wall synthesis and leading to cell lysis and death), aztreonam (also interferes with cell wall synthesis; action against *Pseudomonas aeruginosa*), aminoglycosides (commonly used in more severe open injuries), fluroquinolones (bactericidal by preventing bacterial DNA replication), vancomycin (used against Gram-positive organisms in penicillin-allergic patients).

Elderly patients might benefit more from a combination of first-generation cephalosporin and a fluoroquinolone [82,83]. The most well-known multi-resistant bacteria are *Staphylococcus aureus*, the “ESKAPE” microorganisms (*Enterococcus faecium*, *S. aureus*, *Klebsiella pneumoniae*, *Acinetobacter baumannii*, *Pseudomonas aeruginosa and Enterobacter species*). Many of these organisms have been reported in orthopedic-associated infections [84,85]. Revision arthroplasty surgery for an infected prothesis is associated with significant costs and morbidity [86]. The cause of nosocomial infections in newborns, severely ill and immune-compromised patients, *S. epidermis* is also frequently isolated from post-surgical infections, especially in association with a dwelling prosthetic device, under which circumstances, together with *S. aureus*, it represents a main causative etiological agent [87].

Bacteria can present resistance to antibacterial agents based on their structural and functional characteristics, which are present as a result of two main mechanisms: (a) inherent; (b) acquired (either through mutations or by acquiring the resistance gene from another bacteria). One way by which bacteria develop antibiotic resistance is the biofilm formation in orthopedic implant-associated infections. Biofilms are resistant to antibiotics for various reasons: (a) certain antibiotics fail to penetrate the full depth of the biofilm; (b) some cells within the biofilm are slowly growing; (c) some cells within biofilms may adopt a distinct and protected biofilm phenotype. In one study, the most common bacteria during primary and revision periprosthetic joint infections (PJIs) were *S. aureus* and coagulase-negative *Staphylococci* (CNS), and most strains were resistant to at least one antibiotic, but worrisome in this case is that the CNS resistance to both methicillin and gentamicin seemed to be higher than that of *S. aureus.* Antibiotic resistant *Staphylococci* were also found in orthopedic patients with loosened or failed hip prostheses, even without the phenotypical manifestation of an infection [84,88].

Considering that native Ti implants had been deemed insufficient in the face of bacterial invasion, as well as the cases where this implant is not efficient enough to produce a new bone, several suggestions have been made regarding possible coating of the implants. One of them is by MoS_2_ nanosheets and these exhibit striking antibacterial properties. Previous studies have reported the ability of the nanosheets to induce ROS-independent oxidative stress on contact with bacteria, leading to deleterious effects on bacteria. Furthermore, another study reported the ability of MoS_2_ sheets to accelerate the consumption of intracellular glutathione in order to destroy the antioxidant stress system of the bacteria [89].

Most surgical site infections are acquired during surgery since most cases are due to airborne bacteria or bacteria present on the skin [90]. Sometimes prosthesis removal and replacement represent the only salvage option to eradicate severe infections [91]. Microbial resistance to a drug can manifest in different ways, including an increased drug efflux, drug inactivation, or modification of the antibiotic target [92]. One other alternative for treating infections is the application of powdered topical antibiotics at the surgical site, as it minimizes the systemic exposure and the adverse effects. Typically, antibiotic-impregnated bones grafts have been used for dead-space management during the treatment of osteomyelitis [93].

Another alternative, to try to prevent bacterial infections, is the use of antibiotic-loaded bone cement (ALBC), which has been successful in joint arthroplasty. ALBC presents some advantages over the traditional ways of delivering antibiotics, such as the fact that it allows the use of high concentrations of antibiotics, but with reduced systemic side effects. However, the use of ALBC does not protect against the development of antibiotic resistance in bacteria, as it has an optimal surface for colonizations, and chronic exposure to sub-inhibitory levels allows for mutational resistance to appear [94,95].

Incision infections can be determined by several risk factors, such as local soft tissue injury, surgical bleeding, and long operation duration, and they are still the most common complication after surgery. Most of the times, the infections are mixed, caused by two or more pathogens, with the most common ones being *S. aureus* and *Pseudomonas aeruginosa* in the wounds of patients with extensive burns or complex conditions [96,97].

## 7. COVID-19 and Antimicrobial Resistance

Even if it appears to be a neglected cause of antibacterial resistance, COVID-19 might turn out to be one of the major factors contributing to even higher rates of antimicrobial resistance in the future [98,99,100]. It was revealed that, during the first 6 months of the pandemic, approximately 75% of COVID-19 patients received antimicrobial treatment when only about 8% were confirmed with bacterial or fungal co-infections [101,102].

Professionals have stated that it is hard to differentiate between COVID-19 and bacterial pneumonia co-infection associated with influenza, but COVID-19 is not influenza and a significant proportion of the patients received antimicrobial treatment without confirmed associated secondary infections [103,104]. Even if the symptomatology is very similar to other respiratory diseases, there are still certain symptoms that help the differentiation [105].

A study has found an association between co-infections with Gram-positive bacteria and central venous catheter implantation [106]. Some studies suggest *Candida* sp. as a superinfection associated with COVID-19 and the potential harm of antifungal therapies [107]. Among the patients confirmed with secondary bacterial infections, the most common confirmed bacteria so far included: *Klebsiella*, *Methicillin-susceptible* and *Methicillin-resistant Staphylococcus aureus*, *E. coli, Enterobacter*, *S. pneumoniae,* and *P. aeruginosa* [108]. Interestingly, the majority of these species are also frequently encountered in the case of orthopedic surgeries, as mentioned in the previous paragraph, which suggests that hospital admission might ease the transmission of bacteria, infection, and multi-resistant bacteria, especially when the immune system is weakened. Before the pandemic, world health organizations were on a path of reducing antimicrobial resistance, but in the context of the current world health threat, long-time effects in other areas might be seen as well, such as the antimicrobial resistance reducing significantly the efforts made from the pre-pandemic level [109,110].

Interestingly, one study highlights that patients with severe forms of COVID-19 were more prone to co-infections with bacteria, fungi and other viruses, whereas the mortality rates associated with co-infections were significantly higher for people in the intensive care unit (ICU) or transferred from the ICU to the general wards [111,112]. Rates of resistant bacteria in ICUs are different based on countries, as well as the type of bacteria encountered; however, a study has reported that the number of COVID-19 associated co-infections in ICUs is almost double that of general hospitalized COVID-19 patients [113].

## 8. Conclusions

Thus, as highlighted in this review, the implications of oxidative stress have been described in orthopedic conditions and also in COVID-19. Their separate connections to antibiotic resistant bacteria have also been examined. Several studies have reported higher risks of infections and severe outcomes in patients with rheumatoid arthritis, osteoarthritis, bone fractures and bone replacement surgeries. The major risk factor identified in all of the mentioned disorders is type 2 diabetes and obesity. We have also illustrated that certain drugs can negatively impact the outcome of SARS-CoV-2 infection, even if the said treatment is essential for the management of the underlying condition, e.g., RA. As mentioned by several authors, antibiotic resistance might become an even bigger problem, by prolonging itself in the post-pandemic era, and by becoming even more difficult to counteract. Therefore, there is an acute need for the specialists involved in the field to collaborate and to establish clearer treatment guidelines; ultimately, to avoid any administration of antibiotics when no secondary co-infection is present. Unfortunately, we were unable to identify studies that approach the impact of the mal use of antibiotics during the pandemic in other medical conditions. We consider, however, that it is a topic of interest. Future studies should focus on the manifestations of orthopedic conditions and COVID-19 together and how this impacts the patients: whether the virus worsens the orthopedic conditions or if the latter increases all kinds of risks related to COVID-19, as some very recent studies have reported that post-COVID-19 complications manifest even through conditions such as inflammatory reactive arthritis, joint muscle pain, hypocalcemia and bone demineralization, even in the young population.

## Figures and Tables

**Table 1:** Orthopedic conditions and the risks associated with COVID-19 infection.

Risks	RA	Osteoarthritis	Osteoporosis and Bone Fractures	Diabetes and MetS	Medication for Orthopedic Conditions
**Risk of COVID-19 infection**	↑ [26,27,28,29,30] No association [31]	No apparent association [26]	N.D.	↑ [32] No sign. difference [33]	Systemic therapies no increasing risk [34] ↑ risk of severe infection with GC [27,35] ↑ risk of infection in RA patients with diabetes and treatment with prednisolone and TNFis [36] ↑ risk of infection in RA patients [30] ↑ risk of infection if prior treatment with GC in a dose-dependent manner [26]
**Risk of hospitalization**	↑ [27,28,29,35]	N.D.	↑ risk with a history of hip [37], spine, humerus and wrist fractures [38] ↑ [39]	↑ [40]	N.D.
**Risk of ICU admission**	↑ [35,41]	N.D.	↑ risk for hip fracture patients [37]		N.D.
**Risk of Mechanical ventilation**	↑ [41]	N.D.	↑ in patients with VFs [42] ↑ [39]	↑ [43]	
**Risk of mortality**	↑ [27,28,29,35]	N.D.	↑ risk with a history of hip [44], spine, humerus and wrist fractures [37,38,45,46] ↑ in patients with severe VFs [42] ↑ risk of postoperative mortality in hip fracture population [47] Osteoporotic upper hip fractures—COVID-19 did not modify significantly the 30-day and 6-month mortality [48]	↑ [40,43,49]	At-home glucose-lowering drugs showed no sign. Association with mortality and adverse outcomes [50] -
**Severe outcomes**	↑ [34,51] No association [31]	N.D.	↑ [39]	↑ [33,52,53,54] ↑ increased risk with diabetic ketoacidosis [55] ↑ increased risk with newly diagnosed diabetes and hyperglycemia [56,57]	N.D.
**Other risks associated with COVID-19**	↑ risk of venous thromboembolism and sepsis [58]	N.D.	↑ risk of vertebral fractures among patients with severe SARS-CoV-2 [42] ↑ bone resorption and bone loss [59] ↑ risk of perioperative complication [37]	Diabetes in pediatric population [32], in general population [40] ↑ pneumonia [40] ↑ rates of orthopedic symptoms during the infection [60]	N.D.
**Post-COVID-19 complications**		Inflammatory reactive arthritis [61] Early onset of arthritis, cured by NSAIDS [62] Joint and muscle pain, hypocalcemia, bone demineralization [62]		Type 1 Diabetes [63]	N.D.

N.D.—not determined, GC—glucocorticoids, RA—rheumatoid arthritis, TNFis—tumor necrosis factor inhibitors, VFs—venous fibrillation, NSAIDs—Non-steroidal anti-inflammatory drugs.

## Data Availability

All data is present and available in the current study.
